# A Review of Newborn Screening for VLCADD: The Wisconsin Experience

**DOI:** 10.3390/ijns11020023

**Published:** 2025-03-26

**Authors:** Breanna Mitchell, Jessica Scott-Schwoerer, Ashley Kuhl, Kristina Garcia, Patrice Held

**Affiliations:** 1 Department of Clinical Genomics, Mayo Clinic Health System, Eau Claire, WI 54720, USA; 2 Department of Pediatrics, Medical College of Wisconsin, Milwaukee, WI 53266, USA; jscottschwoerer@mcw.edu; 3 Department of Pediatrics, University of Wisconsin–Madison, Madison, WI 54792, USA; aekuhl@pediatrics.wisc.edu; 4 Academic Affairs, School of Medicine and Public Health, University of Wisconsin, Madison, WI 53705, USA; kgarcia@uwhealth.org; 5 Carbone Cancer Center, UW Health, Madison, WI 53792, USA; 6 Northwest Regional Newborn Screening Program, Hillsboro, OR 97124, USA; patrice.k.held@oha.oregon.gov

**Keywords:** VLCADD, acylcarnitine, newborn screening

## Abstract

Very-long-chain acyl-CoA dehydrogenase deficiency (VLCADD) is due to a defect in metabolism of long-chain fatty acids. Infants with VLCADD may experience cardiomyopathy, hypoglycemia, or even death; thus, early detection and intervention is crucial. The spectrum of disease and natural variation in newborn metabolism, however, lead to overlap in acylcarnitine values between affected and unaffected individuals, which contributes to the difficulty in identifying true positive cases while minimizing false positive cases. VLCADD was added to the state of Wisconsin’s newborn screening (NBS) panel in 2000. A previous retrospective review of VLCADD screen positive cases identified between 2000 and 2014 resulted in a change to the screening algorithm. Following implementation, a reduction in the percentage of false positive screens from 25.3% to 20.4% was observed between 2015 and 2021. The overall PPV also decreased, from 37.2% to 28%, due to an increase in the number of carriers identified (27.5% of cases in 2000–2014 and 51.8% of cases in 2015–2021). A data review also identified three long-chain acylcarnitine elevations (C14:1, C14:1/C16, and C14:1/C2) that had statistically significant differences in concentrations in true positive populations versus false positive populations. Utilization of the C14:1, C14:1/C16, and C14:1/C2 values in newborn screening may provide clearer distinction between true positive and carrier populations and additionally increase the PPV of this screen.

## 1. Introduction

The implementation of newborn screening (NBS) world-wide has improved outcomes for patients born with genetic diseases if acted upon within the neonatal or early childhood stages of life. In the United States, the Advisory Committee on Heritable Disorders in Newborns and Children is a federal committee that has established a recommended uniform screening panel (RUSP) which includes 35 core and 26 secondary disorders [1]. While this RUSP is recommended for every newborn screening program in the United States, it is ultimately up to each individual state to determine which disorders are on their panel. The RUSP also does not specify recommended cutoff values for reporting ‘screen positive’ cases, which can lead to a range of newborn screening practices.

VLCADD (OMIM #201475) is an inborn error of metabolism of long-chain fatty acids. It is an autosomal recessive condition caused by pathogenic variants in the *ACADVL* gene. Symptoms of VLCADD may present in the neonatal or early childhood period as cardiomyopathy, hypoglycemia, lethargy, muscle pain or weakness, and/or exercise intolerance. Patients may also present with periodic rhabdomyolysis in adulthood [2]. Detecting VLCADD in the newborn period is critical as an affected individual may face long-term health consequences or potentially death if the condition is not identified and immediately treated. The addition of VLCADD to newborn screening panels has demonstrated the spectrum of disease symptoms and severity, including a subset of patients with very mild or asymptomatic presentation [2]. It is unclear if these mildly affected populations represent a true spectrum of disease or are a result of early detection and intervention.

VLCADD screening was added to the newborn screening panel in Wisconsin in 2000, and it is currently screened for in all 50 states via tandem mass spectrometry. While there is a clear need for and demonstrated benefit to detecting VLCADD in the newborn period, the screening and reporting processes are not consistent from state to state. Assay performance, reporting parameters, and variation in populations can lead to differences in screen positive cutoff values across the screening programs. Additionally, the spectrum of disease as well as natural variations in newborn metabolism contribute to the difficulty in identifying a screening cutoff value that minimizes false positives and maximizes true positives.

Confirmatory testing for VLCADD comes with its own challenges as well. Beyond the ACMG recommendation of confirmatory metabolic labs including a plasma acylcarnitine profile, providers may order *ACADVL* gene sequencing and/or functional enzyme or fatty acid oxidation assays. The results of this testing may, at times, lead to conflicting or uncertain results in borderline cases [3]. DNA testing may result in identification of a variant of uncertain significance (VUS) or be limited by methods that may not detect a second variant, and VLCADD and carrier populations have been demonstrated to have some overlap in residual enzyme activity [4]. Confirmatory testing procedures vary from state to state and may not be consistent even between various metabolic clinics within the same state, which may lead to inconsistent diagnoses.

In 2013, the Western States Regional Genetics Services Collaborative performed a retrospective analysis on VLCADD screen positive results from California, Washington, Oregon, and Hawaii with the aim to develop more effective diagnostic algorithms. Their study identified the usefulness of C14:1, C14, C14:1/C2, and C14:1/C16 values between affected individuals and carriers/false positives. Their positive predicted values (PPVs) for true positives were 94%, 54%, and 23% when C14:1 was ≥2.0 μM, ≥1.0 μM, and ≥0.7 μM, respectively [5]. Similarly, in 2016, Atkins et al. implemented a confirmatory testing protocol for VLCADD screen positive cases in Iowa to determine if case classification could be improved. As a result of this study, case classifications were simplified and streamlined using consistent follow-up protocols [6]. Both of these studies demonstrate the need for quality improvement in VLCADD newborn screening and confirmatory testing procedures.

In 2014 the Wisconsin State Laboratory of Hygiene (WSLH) reviewed 51 abnormal VLCADD newborn screen results from 2000 (when VLCADD was added to the screen) to 2014 to determine which values and ranges most appropriately identified true positive screens and minimized false positives [7]. As a result of this study, WSLH implemented new criteria for determining a screen positive VLCAD deficiency case. The historical algorithms are outlined in Table 1.

The purpose of this study is to review and analyze VLCADD screen positive results identified in the Wisconsin newborn screening program from 2015 to 2021. Evaluation of these data involves assessing the effectiveness of the protocol changes implemented in 2014 for reducing false positive screens while appropriately capturing affected individuals.

## 2. Materials and Methods

Biochemical data for all infants born in Wisconsin who screened positive for VLCADD were provided by WSLH. These data included all reported acylcarnitine values from the individuals’ newborn screening cards, which were selected by the Wisconsin State Lab of Hygiene based on laboratory best practices and a literature review. Multiple repeat acylcarnitine profiles were performed on all initial VLCADD screen positive patients. The acylcarnitine values used in this study were the values reported to the patients’ providers and may not have all been derived from the same test run. Patients were referred for follow-up with genetics providers at either the University of Wisconsin Health American Family Children’s Hospital or Children’s Wisconsin. Follow-up testing included a combination of biochemical testing including an acylcarnitine profile and urine organic acids, molecular testing of the gene *ACADVL*, a fatty oxidation probe, and/or VLCAD enzyme (fibroblast or white blood cell) testing. Due to concerns about normalization of acylcarnitine profiles with improved clinical status, i.e., decreased catabolism with improved feeding, patients did have at a minimum molecular or enzyme/probe studies, oftentimes both. Clinical practice remained similar throughout the study.

Medical records of the identified patients were reviewed by the study team to confirm clinical outcomes and clarify cases that were lost to follow-up, did not have a conclusive result reported to the State Lab of Hygiene, or had a reclassification of diagnosis. Patients who ultimately did not have a resolution were not utilized in this data review. In this study, the type of confirmatory testing that led to the designation of true positive, carrier, or unaffected was not tabulated. Sensitivity, specificity, and positive predictive values were calculated for the groups of screen positive cases between 2000 and 2014 and 2015 and 2021.

Concentrations of each acylcarnitine value were summarized using the median and interquartile range (IQR) and separated according to final diagnosis. The Kruskal–Wallis test was used to address whether concentrations varied by diagnosis, and pairwise comparisons were performed using the Steel–Dwass multiple comparison procedure to determine if the initial test supported a difference [8]. All analyses were performed using R (version 4.1.1) [9]. Data for cases lost to follow-up and for cases ultimately determined to have a different diagnosis were not included in these analyses.

## 3. Results

Between 2000 and 2014, there were 51 newborns who screened positive for VLCADD. The data analyzed in the 2014 review initially classified 21 of these cases as VLCADD and 12 as false positive carriers. Re-review of patient charts within UW Health determined two VLCADD cases had since been reclassified as false positive carriers due to updated enzyme testing. The updated PPV with these reclassifications was 37.3%. Between 2015 and 2021, there were 57 newborns with screen positive results for VLCADD. Six cases initially did not have a resolution; however, following chart review three of these six cases were able to be classified, leaving three without a resolution. Case outcomes and test performance are categorized in Table 2. Using the 54 cases with final outcomes, the PPV for this period is 27.8%.

When evaluating the three disease outcome groups individually (VLCADD, false positive carrier, and false positive with no mutation), we identified that the proportion of false positive with no mutation cases initially flagged as abnormal by newborn screen decreased from 35.3% to 20.4%, while the number of false positive carriers identified increased from 27.4% to 51.8%, leading to an overall reduced PPV.

To the knowledge of the study team, there are no documented cases of patients diagnosed with VLCADD outside of newborn screening (i.e., missed cases). For this reason, the sensitivity and negative predictive value for both periods are 100%. The specificity for both periods was calculated to be 99.9%. The VLCADD disease incidence between 2000 and 2014 was 1/54,000, and the disease incidence between 2015 and 2021 was 1/30,000, providing an overall disease incidence of 1/43,000 in Wisconsin since the inception of VLCADD in newborn screening.

The distributions of C14:1, C14:1/C16, and C14:1/C2 values were found to be statistically significantly different (*p* = 0.001, *p* = 0.001, and *p* = 0.007, respectively) between the affected, false positive carrier, and false positive with no mutation populations (Table 3). The C14:1 values in individuals affected by VLCADD were significantly different in comparison to both the false positive carrier group (*p* = 0.008) and the false positive with no mutation group (*p* = 0.005). There was no significant difference in C14:1 values between the false positive carrier and false positive with no mutation groups (*p* = 0.165).

The ratios of C14:1 to C16 and C14:1 to C2 were also identified to be statistically significantly different (*p* < 0.001 and *p* = 0.007, respectively) among the three populations. Both ratios in affected individuals were significantly different from the false positive groups (*p* ≤ 0.027). To some degree, the significance of both ratios is due to the initial significance of the C14:1 acylcarnitine acting in the numerator.

## 4. Discussion

While the criteria implemented in 2014 were effective in reducing the proportion of cases without mutations, it is concerning that the proportion of screen positive carriers significantly increased. It is well established that there is a high degree of overlap in acylcarnitine profiles between affected and carrier populations [4]. While the modified criteria may rule out more false positives with no mutations, they may not be able to distinguish between carriers and affected individuals.

Reviewing the analysis of the acylcarnitine values and ratios compared to the current screening criteria, we can identify similarities and differences. C14:1 and C14:1/C16 values were statistically different between the VLCADD and the false positive populations (Table 2). However, there are demonstrated gaps between the screening rules and data values. For example, the averages and lower IQR bounds of the C14:1 and C14:1/C16 values for affected individuals were all above the current screen positive thresholds of 1.0 and 0.25, respectively. This suggests that more stringent criteria may be warranted to determine the screen positive thresholds of these values. Additionally, the [C14 or C14:2] rule did not yield statistical significance in these values between VLCADD and carrier/false positive individuals. There was, in fact, very high overlap in these values between individuals with VLCADD and false positive carriers (Table 2), which may have contributed to the increased proportion of false positive carriers identified between 2015 and 2021.

The data identified a third statistically significant ratio: C14:1/C2. The three values identified as significant in this study (C14:1, C14:1/C16, and C14:1/C2) are highly consistent with the previously mentioned Western States Regional Genetics Services Collaborative study which identified the importance of C14:1, C14, C14:1/C2, and C14:1/C16 values [5]. While the C14:1/C2 value is not currently part of the Wisconsin newborn screening rules, considering the significance in the data set and consistency in significance with other studies, it may be appropriate to consider it as a potential modification to the third rule of the current criteria. Implementing a rule of C14:1/C2 ≥ 0.40 on the 2015–2021 data would have prevented a screen positive report for 15 of the 28 false positive carriers and 7 of the 11 false positive no mutation cases that had been identified, as well as only 1 individual with VLCADD (who also had a C14:1 value below the current threshold).

While not statistically significant, the analysis did identify an unusual correlation between elevation of C16:1OH and disease status. On average, C16:1OH values were higher among false positive individuals compared to those identified to have VLCADD. This goes against accepted knowledge that individuals with VLCADD have higher elevations of long-chain fatty acids compared to their carrier and unaffected counterparts. It is unknown why this counter-correlation was identified, but it may be relevant to investigate it in other populations and further studies. A previous study has suggested that antibiotics (ampicillin, cefotaxime) interfere with C16:1OH levels [10]. While this study does not include data on antibiotic use in the individuals in our data set, inclusion of these data may be helpful in future assessments.

The five-year post-2014 data review identified only four fewer individuals with VLCADD compared to the previous 14 years, indicating an increase in disease incidence in Wisconsin. The overall disease incidence from 2000 to 2021 (1/43,000) is, however, in line with what the literature suggests (1/40,000) [11]. Interestingly, the Netherlands also noted an increase in disease prevalence following the addition of VLCADD to their newborn screen in 2007; from 2007 (to publication in 2018), the disease incidence was reported as 1:55,000, compared to 1:350,000 prior to 2007 [12]. These data support previous suggestions that we are detecting more individuals with VLCADD who have very mild symptoms or no symptoms at all and would have otherwise gone undiagnosed. The increase in disease incidence could also correlate to the increase in the number of carriers identified on screening.

## 5. Conclusions

The 2014 criteria were effective in reducing false positive cases, indicating unaffected individuals have greater differences in biochemical profiles compared to carriers and affected individuals. The number of false positive carriers picked up by screening increased, however, which led to a lower overall PPV of the screen and suggests additional modification to the criteria may still be needed.

C14:1 and C14:1/C16 values continue to be highly correlated with disease status; per the data analysis, it may be reasonable to consider increasing the cutoff for these values to reduce the number of false positive carriers identified on the screen. Individuals with VLCADD also had C14:1/C2 values that were significantly different from the false positive populations. These values did not show statistical differences between the false positive carrier and false positive no mutation populations, suggesting that the C14:1, C14:1/C16, and C14:1/C2 values may be most indicative of the VLCADD population.

## Figures and Tables

**Table 1:** Summary of VLCADD algorithm changes from inception in 2000 to 2014. The 2004 algorithm reflects the addition of the C14:1/C16 ratio. The 2007 algorithm reflects the removal of C16. In 2014, cutoff values were adjusted based on a retrospective review of previous results.

Year	Normal	Possibly Abnormal Repeat Sample	Abnormal
2000	C14:1 < 0.6 μmol/L	C14:1 > 0.6 μmol/L	C14:1 > 0.6 μmol/L AND C14 > 0.8 μmol/L AND C14:2 > 0.18 μmol/L AND C16 > 0.23 μmol/L
2004	C14:1 < 0.6 μmol/L C14:1/C16 < 0.25	C14:1 > 0.6 μmol/L C14:1/C16 > 0.25	C14:1 > 0.6 μmol/L AND C14:1/C16 > 0.25 C14 > 0.8 μmol/L AND C14:2 > 0.18 μmol/L AND C16 > 0.23 μmol/L
2007	Same as 2004	Same as 2004	C14:1 > 0.6 μmol/L AND C14:1/C16 > 0.25 C14 > 0.8 μmol/L AND C14:2 > 0.18 μmol/L
2014	C14:1 < 1 μmol/L C14:1/C16 < 0.25	N/A	C14:1 > 1.0 μmol/L AND C14:1/C16 > 0.25 AND Either C14 > 0.8 μmol/L OR C14:2 > 0.18 μmol/L

**Table 2:** Summary of screen positive cases classified by outcome (%) and sensitivity, specificity, and PPV calculations.

Outcome	2000–2014	2015–2021
VLCADD (true positive)	19 (37.3%)	15 (27.8%)
False positive (carrier)	14 (27.4%)	28 (51.8%)
False positive (no mutation)	18 * (35.3%)	11 * (20.4%)
Lost to follow-up	0	3
Total screen positive cases	51	57
Total newborns screened	1,025,876	442,987
Positive predictive value	37.3%	27.8%
Negative predictive value	100%	100%
Sensitivity	100%	100%
Specificity	99.9%	99.9%

* Two cases between 2000 and 2014 and one case between 2015 and 2021 were ultimately determined to have glutaric acidemia II.

**Table 3:** Concentration of each acylcarnitine considered separated by diagnosis and reported as the median and inter quartile range (IQR) obtained from newborn screening of dried blood spot samples. Sample sizes are 10 (false positive), 27 (carrier), and 15 (VLCAD). Comparison among groups was performed using the Kruskal–Wallis (K-W) test with significant values in bold.

Fatty Acid Chain	False Positive No Mutation Med [IQR]	False Positive Carrier Med [IQR]	Affected Med [IQR]	*p*-Value
C12:1	0.42 [0.10, 0.62]	0.52 [0.32, 0.62]	0.51 [0.31, 0.69]	0.940
C12	0.62 [0.21, 0.80]	0.61 [0.50, 0.72]	0.64 [0.47, 0.84]	0.779
C14:2	0.16 [0.09, 0.29]	0.17 [0.14, 0.21]	0.24 [0.13, 0.38]	0.299
**C14:1**	**0.91 [0.48, 1.07]**	**1.08 [0.83, 1.34]**	**1.76 [1.15, 3.41]**	**0.001**
C14	0.74 [0.28, 1.13]	0.91 [0.71, 1.12]	1.28 [0.70, 2.56]	0.143
C14OH	0.19 [0.05, 0.73]	0.06 [0.04, 0.19]	0.06 [0.04, 0.11]	0.374
C16:1	0.51 [0.41, 0.66]	0.05 [0.45, 0.65]	0.67 [0.47, 1.00]	0.190
C16	2.63 [0.41, 0.66]	3.78 [2.69, 4.36]	4.38 [2.92, 5.60]	0.130
C16:1OH	0.36 [0.08, 1.56]	0.10 [0.06, 0.37]	0.08 [0.05, 0.15]	0.222
C16OH	0.15 [0.07, 0.55]	0.08 [0.05. 0.14]	0.08 [0.03, 0.17]	0.432
**C14:1/C16**	**0.22 [0.10, 0.29]**	**0.27 [0.21, 0.34]**	**0.42 [0.34, 0.64]**	**<0.001**
**C14:1/C2**	**0.03 [0.02, 0.07]**	**0.04 [0.03, 0.05]**	**0.06 [0.05, 0.15]**	**0.007**

## Data Availability

The raw data supporting the conclusions of this article will be made available by the authors on request.
